# Knowledge and Information Sources About COVID-19 Among University Students in Jordan: A Cross-Sectional Study

**DOI:** 10.3389/fpubh.2020.00254

**Published:** 2020-05-29

**Authors:** Amin N. Olaimat, Iman Aolymat, Hafiz M. Shahbaz, Richard A. Holley

**Affiliations:** ^1^Department of Clinical Nutrition and Dietetics, Faculty of Applied Medical Sciences, The Hashemite University, Zarqa, Jordan; ^2^Department of Cellular and Molecular Physiology, Faculty of Health and Life Sciences, School of Medicine, University of Liverpool, Liverpool, United Kingdom; ^3^Department of Basic Medical Sciences, Faculty of Medicine, The Hashemite University, Zarqa, Jordan; ^4^Department of Food Science and Human Nutrition, University of Veterinary and Animal Sciences, Lahore, Pakistan; ^5^Department of Food and Human Nutritional Sciences, University of Manitoba, Winnipeg, MB, Canada

**Keywords:** COVID-19, coronavirus, SARS-CoV-2 virus, knowledge, awareness, information source, university student, Jordan

## Abstract

**Background:** Coronavirus disease 2019 (COVID-19) has rapidly spread worldwide, and it was officially declared to be a pandemic by the World Health Organization (WHO) on March 11, 2020. Most countries over the entire globe have reported some COVID-19 cases. The current study aimed to assess student knowledge about COVID-19 at different Jordanian universities and determine where they sourced their information.

**Methods:**A cross-sectional study was conducted among 2,083 undergraduate or postgraduate students from different governmental and private universities during the initial stage of the disease in Jordan (March 19–21, 2020) using a validated, structured, self-administered, online questionnaire. The survey was structured to assess their knowledge about viral sources, incubation period, mortality rate, transmission, symptoms and complications as well as the source of information about COVID-19.

**Results:**Overall, 56.5% of the respondents showed good knowledge and almost 40.5% showed moderate knowledge. On the other hand, 3.0% of the participants showed poor knowledge about COVID-19. The average knowledge score of students was 80.1%, which is considered to be within the scale of good knowledge. Both the college of study and educational level significantly (*P* < 0.05) associated with student knowledge. Students who majored in medical sciences showed the highest mean score of 82.8%, with 69.0% displaying a good knowledge level. Postgraduate students had significantly higher knowledge scores compared to undergraduate students. The majority of students used the internet, social media and mass media as sources of information about COVID-19. Scientific websites and articles were used more commonly by medical and postgraduate students.

**Conclusions:**The COVID-19 pandemic is a major challenge to the health of the world population; therefore, these results assessing students' knowledge provide an important baseline for planning required educational interventions such as contact tracing and self-quarantine. These results may also help public health authorities by engaging communities in implementation of protective health measures, including positive hygienic practices such as hand washing to reduce the risk of COVID-19.

## Introduction

Coronavirus disease 2019 (COVID-19) is an emerging respiratory infection caused by a novel coronavirus called Severe Acute Respiratory Syndrome coronavirus 2 (SARS-CoV-2). The virus is a member of the coronavirus family that are zoonotic pathogens, i.e., the viruses cause and transmit illnesses between human and several animals species such as cattle, camels, cats, and bats (1, 2). The SARS-CoV-2 virus is similar to Middle East Respiratory Syndrome coronavirus (MERS-CoV) and Severe Acute Respiratory Syndrome coronavirus (SARS-CoV), which have their origins in bats. The COVID-19 disease was detected initially in late December 2019 in Wuhan, Hubei Province, China, and spread worldwide 2 months later. About 200 countries over the entire world have reported different numbers of cases; however, the disease has drastically expanded in the United States, Spain, Italy, Germany, France, China, Iran, the United Kingdom, and Turkey. COVID-19 had caused more than 3.7 million confirmed cases and killed at least 260,000 worldwide up to the 11th of April 2020, and these numbers were expected to rise dramatically in the next few months (3). To date, 473 COVID-19-infected cases have been confirmed in Jordan, and 9 people have died with COVID-19 (4).

The symptoms of COVID-19 illness range from very mild (fever and respiratory symptoms such as cough and shortness of breath) to severe (pneumonia, severe acute respiratory syndrome and kidney failure) with a mortality rate around 4% (3). Elderly persons and those suffering from co-morbidities like heart disease, lung disease and diabetes, are at higher risk of developing severe COVID-19 illness. On March 18, 2020, the CDC COVID-19 Response Team reported that 80% of COVID-19-related deaths were among the elderly aged > 65 years (5). As a response to this serious global public threat, the WHO characterized the COVID-19 outbreak as a pandemic on March 11, 2020, since the number of COVID-19 cases outside China had increased by 13-fold, and the number of affected countries had increased by 3-fold (3).

A limited number of *in vitro* and clinical studies have reported that some medications such as chloroquine, hydroxychloroquine, remdesivir and azithromycin have the potential to reduce the duration and symptoms of COVID-19 infection (2, 6–8). Unfortunately, a curative treatment or vaccine for the SARS-CoV-2 virus has not been developed yet, and the available medical interventions are supportive only.

COVID-19 disease has negatively affected global economics, and this has included the Jordanian economy. Furthermore, many healthcare systems have collapsed or nearly collapsed due to COVID-19 (9, 10). Therefore, it is very important to flatten the shape of the crest in case numbers as much as possible while communities experience an outbreak of COVID-19 to reduce the burden on the healthcare system.

In response to lessons learnt from the previous pandemic (H1N1) 2009 virus and SARS in 2003, management measures should be considered. These measures include prevention of the infection within animals, its transmission from animals to humans, and its transmission among humans (11, 12). The latter is highly affected by promoting good hygienic practices among people to include enhancement of hand washing, use of personal protective equipment and minimization of hand-to-face contact (13). During the current pandemic, most countries are responding to contain the COVID-19 pandemic by retarding infection spread using different strategies such as contact tracing and self-quarantine, arrangement of health system infrastructures to treat severely infected patients who need isolation, oxygen therapy or mechanical ventilation, reducing, or banning events involving mass gatherings, and encouraging people to apply hygienic health measures, such as physical distancing, respiratory etiquette and frequent hand washing. The latter strategy requires a high level of knowledge about COVID-19 fostering attitudes among people to recognize and practice these measures properly. In the absence of COVID-19 treatment, the application of protective measures will potentially prevent the population from acquiring the disease and reduce disease dissemination (14, 15). As a result, this study aimed to assess the knowledge and information sources of undergraduate and postgraduate students at different Jordanian universities toward COVID-19 infection. As a youthful country, a large number of young Jordanians are enrolled in a total of 33 governmental and private universities. Thus, their awareness levels will roughly reflect the public knowledge about COVID-19, which will constitute a general reference to guide the local authorities in planning the required educational interventions.

## Materials and Methods

### Sample Size Calculation

In the current study, the estimated sample size was derived from the online Raosoft sample size calculator (16). The sample size was calculated based on a response rate of 50%, a confidence interval of 99%, and a margin of error of 5%, with a total university student population of 377,000. Although the required sample size was 663, in the current study the sample size used was 3-fold larger than that required.

### Study Design and Preparation of Questionnaire

A cross-sectional study was conducted among 2083 government and private university students in Jordan between March 19 and 21, 2020. On March 2, the first confirmed case of COVID-19 was reported in Jordan, and on March 18, the government imposed stringent social/business restrictions for 1 month to contain the disease. The survey questionnaire was initially prepared in English and then translated into Arabic with the assistance of an independent, bilingual, professional translator whose native language was Arabic. The first part of the questionnaire involved an introduction showing the objectives of the study and highlighting that participation in this study was voluntary, and that the answers would be treated confidentially. Participants were not offered any financial compensation. The completion of the online survey took about 8–10 min and included multiple-choice questions, or yes/no/ I don't know options within different sections. A second section determined the socio-demographic variables of the students including gender, age, university location, major field of study, education level, and place and type of residence. The third section measured the students' knowledge about COVID-19, such as its sources, incubation period, mortality rate, transmission, symptoms, and complications. Another section explored the source they used for information about COVID-19. The remaining survey sections, including information about the attitudes and practices of students regarding COVID-19 infection have been submitted for publication elsewhere.

### Consistency and Validation of Questionnaire

The questionnaire was prepared based on the available information on the web sites of the European and American Centers for Disease Control (ECDC and CDC, respectively) and the World Health Organization (WHO). The questionnaire was reviewed by a panel of experts and revised based on their comments. After that, a pilot study was conducted to evaluate the internal consistency and validity of the Arabic version of the questionnaire by asking 29 students to complete the translated questionnaire. Cronbach's alpha was calculated, and it was within the acceptable level (≥0.70) with a value of 0.74.

### Ethics Approval

The survey project was evaluated and approved by the Institutional Review Board (IRB) committee of the Hashemite University. Further, an informed consent form was obtained from all participants prior to their participation in the study showing that involvement in the completion of the questionnaire was voluntary, that students were able to withdraw at any stage of the survey, and that their answers would be treated confidentially.

### Data Collection Procedure

Data were collected using a self-administered, online survey via Google forms due to the complete lockdown of Jordan. Students were invited to complete an anonymous online survey through Facebook and WhatsApp groups of university students.

### Statistical Analysis

The collected data were analyzed using the Statistical Package of Social Sciences software (SPSS) version 25. A system of question scoring was used to measure the level of students' knowledge by giving a score of 1 for the correct answer and 0 score for an incorrect or I don't know answer for each question. The total score of students' knowledge was converted to a percentage, over a range of 0 to 100%. The knowledge scores were classified a spoor ( ≤ 60%), moderate (60.01–80%), and good knowledge (≥80.01%). The results of students' knowledge of COVID-19 and sources of information were expressed as frequencies and percentages. The results were analyzed using analysis of variance (ANOVA) and Pearson's Chi square test (X^2^) was used to illustrate the statistical differences among the categories of socio-demographic variables. Cronbach's alpha was calculated to assess the internal consistency of the questionnaire. Statistically significant differences were considered when *P* < 0.05.

## Results

### Demographic Characteristics and Knowledge Score of COVID-19 Among Participants

Of the 2,083 student participants in this study almost three quarters, or 1,572, were female. Undergraduate students represented the majority of the participants at 90.2%, with the remaining 9.8%, being postgraduate students. The proportion of participants according to their majors were as follows: 415 students (19.9%) were from the engineering school, 535 students (25.7%) were from the medical sciences school, 376 students (18.1%) were from the agriculture and general sciences school and 757 students (36.3%) were from the human sciences school. Among the participants, 498 (23.9%), 1,304 (62.6%), and 281 (13.5%) students were within the ages of 18–19.9, 20–24.9, and ≥25 years, respectively. Other demographic variables such as university location, place of residence and type of accommodation are presented in Table 1.

**Table 1:** Demographic characteristics of university students and knowledge score of COVID-19 among University students in Jordan by socio-demographic variables.

**Demographic variable**	**Category**	**N**	**Knowledge category (%)**			**Knowledge score (%)**	***X*^**2**^**	**P-value**
			**Poor**	**Moderate**	**Good**			
Overall			62 (3.0)	844 (40.5)	1,177 (56.5)	80.1		
Age	18–19.9 years	498	19 (3.8)	211 (42.4)	268 (53.8)	79.2	5.414	0.247
	20–24.9 years	1,304	36 (2.8)	532 (40.8)	736 (56.4)	80.2		
	≥25 years	281	7 (2.5)	101 (35.9)	173 (61.6)	81.6		
Gender	Female	1,572	48 (3.1)	626 (39.8)	898 (57.1)	80.3	1.330	0.514
	Male	511	14 (2.7)	218 (42.7)	279 (54.6)	79.7		
University location	Northern universities	551	13 (2.8)	208 (37.7)	330 (59.9)	81.1	4.546	0.337
	Middle universities	1,477	48 (3.2)	611 (41.4)	818 (55.4)	79.9		
	Southern universities	55	1 (1.8)	25 (45.5)	29 (52.7)	78.2		
College	Engineering	415	7 (1.7)	167 (40.2)	241 (58.1)	80.3	77.738	0.000
	Medical sciences	535	8 (1.5)	158 (29.5)	369 (69.0)	82.8		
	Agriculture and general sciences	376	9 (2.4)	147 (39.1)	220 (58.5)	80.5		
	Human sciences	757	38 (5.0)	372 (49.1)	347 (45.8)	78.0		
Education level	Undergraduate	1,879	54 (2.9)	778 (41.4)	1,047 (55.7)	80.0	6.487	0.039
	Postgraduate	204	8 (3.9)	66 (32.4)	130 (63.7)	81.9		
Accommodation type	Villa	106	2 (1.9)	39 (36.8)	65 (61.3)	80.7	9.185	0.163
	Flat	798	22 (2.8)	298 (37.3)	478 (59.9)	80.8		
	House	1,153	37 (3.2)	494 (42.8)	622 (53.9)	79.7		
	Others	26	1 (3.8)	13 (50.0)	12 (46.2)	78.5		
Place of residence	City	1,642	47 (2.9)	653 (39.8)	942 (57.4)	80.3	4.015	0.404
	Village	390	13 (3.3)	165 (42.3)	212 (54.4)	79.8		
	Others	51	2 (3.9)	26 (51.0)	23 (45.1)	78.2		

In general, more than half of the respondents (56.5%) showed good knowledge of COVID-19. The proportion of university students who showed moderate knowledge of COVID-19 was 40.5%. Only a small proportion (3.0%) of the participants showed poor knowledge of COVID-19. The average score of respondents was 80.1%, which is considered to be good. Socio-demographic variables, including age, gender, university location, accommodation type and place of residence were not significantly (*P* > 0.05) associated with the knowledge score. However, it was noticed that the percentage of the students with a good knowledge score increased as age increased. Approximately, 53.8, 56.4, and 61.6% of the students in age categories of <20, 20–24.9 and ≥25 years, respectively, had good knowledge with a mean score that ranged from 79.2 to 81.6%. Both the college of study and educational level significantly (*P* < 0.05) associated with student knowledge of COVID-19. Students who majored in medical sciences showed the highest mean knowledge score, 82.8%, with 69.0% of the students in this discipline showing a good knowledge level. This was followed by students of agriculture and general sciences and engineering who showed a similar knowledge score (around 80%). On the other hand, the students of human sciences showed the lowest average knowledge score, which was 78.0%. It was notable that the postgraduate students had a significantly higher score of 81.9% compared to the 80.0% of the undergraduate students. Furthermore, 63.7% of the postgraduate students had a good knowledge level compared to 55.7% of the undergraduate students (Table 1).

### Detailed Responses of University Students About COVID-19

The vast majority (96.3%) of the university students had heard about COVID-19. The knowledge of the students about COVID-19 is detailed in Tables 2, 3. Among the 12 questions assessing the general awareness of COVID-19, 7 questions were correctly answered, with percentages ranging between 85.4 and 99.4%. These questions evaluated the students' knowledge about cause, incubation period of COVID-19, the need for isolation and emergency or curative treatment of infected persons, and the presence of infected individuals in Jordan. Further, 71.0–72.7% of the participants recognized that COVID-19 is caused by a novel member of the coronaviruses, and that there is no effective medication or vaccine for its control. About 59.1% of the students were aware that the approximate mortality rate of COVID-19 is ≤ 5%. Additionally, about one-third of the respondents (34.6%) expected that genetic material of the virus was DNA. The percentage of “don't know” answers in these questions increased as the students' knowledge decreased and ranged from 0.3 to 46.7% (Table 2).

**Table 2:** Responses about general knowledge of COVID-19 among University students in Jordan.

**Question (Correct answer)**	**Correct answers**		**Incorrect answers**	
	**N**.	**%**	**N**.	**%**
The cause of the COVID-19 disease is Virus (Yes)	1,961	94.1	122	5.9
The type of genetic material in COVID-19 is DNA (No)	720	34.6	1363	65.4
COVID-19 is caused by a new member of coronavirus (Yes)	1,478	71.0	605	29.0
Presence of COVID-19 cases in Jordan (Yes)	1,995	95.8	88	4.2
COVID-19 cases should be immediately isolated (Yes)	2,071	99.4	12	0.6
Antibiotic is an effective medication in the treatment of COVID-19 (No)	1,495	71.8	588	28.2
Most COVID-19 infected people can recover completely (Yes)	1,849	88.8	234	11.2
There is vaccine for COVID-19 (No)	1,515	72.7	568	27.3
There is no effective curative treatment for COVID-19 (Yes)	1,779	85.4	304	14.6
Intensive and emergency treatment should be given to diagnosed patients (Yes)	1,790	85.9	293	14.1
Generally, incubation period for COVID-19 is ≤ 14 days (Yes)	1,964	94.3	119	5.7
The approximate mortality rate of COVID-19 is > 5% (No)	1,232	59.1	851	40.9

**Table 3:** Responses about knowledge of COVID-19 transmission, symptoms, complications and people at high risk among university students in Jordan.

**Question (Correct answer)**	**Correct answers**		**Incorrect answers**	
	**N**.	**%**	**N**.	**%**
**Mode of COVID-19 transmission includes:**				
Saliva and nasal drip from the sick COVID-19 patient (Yes)	1,695	81.4	388	18.6
Coughing and sneezing (Yes)	1,877	90.1	206	9.9
Touching the nose or mouth (Yes)	1,812	87.0	271	13.0
Kissing and shaking hands (Yes)	1,973	94.7	110	5.3
The use of objects owned by an COVID-19 infected person (Yes)	1,807	86.7	276	13.3
Touching contaminated surfaces (Yes)	1,962	94.2	121	5.8
Consuming foods (No)	1,600	76.8	483	23.2
Sexual route (No)	1,352	64.9	731	35.1
Air (No)	1,497	71.9	586	28.1
**People who are vulnerable to develop complications include:**				
Adults (No)	1,871	89.8	212	10.2
Children less than 5 years old (No)	1,251	60.1	832	39.9
People with co-morbidity such as diabetes, cancer and other chronic diseases (Yes)	1,687	81.0	396	19.0
Elderly (Yes)	1,970	94.6	113	5.4
**The symptoms of the disease may include:**				
Fever (Yes)	1,939	93.1	144	6.9
Blurred vision (No)	1,945	93.4	138	6.6
Dry cough (Yes)	1,917	92.0	166	8.0
Myalgia (Yes)	923	44.3	1160	55.7
Sore throat (Yes)	1,536	73.7	547	26.3
Runny nose (Yes)	841	40.4	1242	59.6
Difficulty breathing (Yes)	1,877	90.1	206	9.9
Skin rash (No)	2,047	98.3	36	1.7
Diarrhea (Yes)	850	40.8	1233	59.2
Vomiting (Yes)	602	28.9	1481	71.1
**Complications of COVID-19 infection include:**				
Pneumonia (Yes)	1,840	88.3	243	11.7
Sepsis (Yes)	95	4.6	1988	95.4
Bronchitis (Yes)	1,649	79.2	434	20.8
Neuropathy (No)	1,929	92.6	154	7.4
Multi-organ failure (Yes)	1,150	55.2	933	44.8
Hyperglycemia (No)	1,958	94.0	125	6.0
Severe illness with respiratory failure can lead to death (Yes)	1,899	91.2	184	8.8

Overall, the students showed moderate to good knowledge of the transmission mode of COVID-19. Correct answers for the mode of transmission question ranged from 64.9 to 94.7%. Most of the students were aware that elderly (94.9%) and immunocompromised persons (81.0%) are at higher risk to develop severe cases of COVID-19. Further, 89.8% and 60.1% of the students realized that healthy adults and children, respectively, are not at higher risk for severe illness. The majority of the students correctly answered that fever (93.1%), dry cough (92.0%) and shortness of breath (90.1%) are among the most commonly reported symptoms of COVID-19. About three quarters of the students were aware that sore throat is one of the COVID-19 symptoms. On the other hand, the students showed poor knowledge about other symptoms which can be reported in a few people including myalgia (44.3%), rhinorrhea (40.4%), diarrhea (40.8%) and vomiting (28.9%). Furthermore, the vast proportion of students knew that blurred vision (93.4%) and skin rash (98.3%) are not normally symptoms of COVID-19. A major portion of the students (91.2%) knew that severe illness from COVID-19 can lead to death. The students also showed good knowledge in recognizing that pneumonia (88.3%) and bronchitis (79.2%) are complications of COVID-19. However, 55.2% of the students recognized that COVID-19 could cause damage to some organs such as the kidney, liver and heart. On the other hand, only 4.6% of the students were aware that sepsis could complicate COVID-19 in some cases (Table 3).

### Source of Information

The most common source of the students' information about COVID-19 was the internet (1605, 77.1%), including electronic news websites and social media such as Twitter, Facebook, YouTube, Instagram, Snapchat and WhatsApp, followed by mass media (1,408, 67.6%) such as TV, newspapers, magazines, and radio, and then scientific websites and articles (505, 24.2%). A very small proportion the participants (145, 7.0%) obtained their information from other sources such as friends and family. There were no significant differences among categories of each student demographic for use of mass media as a source of information. Gender, college, accommodation type and place of residence significantly (*P* < 0.05) associated with the use of the internet and social media as a source of information among the university students. About 81.8% of males used social media as a source of information compared to 75.5% of females. Among the college of study, engineering students (84.3%) were the uppermost group, who used social media for their information about COVID-19. By contrast, students of human sciences were the group least likely to use social media for information regarding COVID-19. The study also revealed that the majority of students (78.5%) who live in cities obtained their information about COVID-19 from social media compared to their counterpart in villages (72.3%) or other places of residence (66.7%) such as camps. Acquisition of information from scientific websites and articles was significantly (*P* < 0.05) affected by age, gender, university location, college of study and education level. A proportional relationship between age and obtaining information from scientific websites and articles was observed in this study. Moreover, males (30.7%) used scientific websites and articles significantly more than the females (22.1%). Among different colleges, students of medical sciences (33.1%) used scientific websites and articles significantly more than other students. Only 17.0% of human sciences students used scientific websites and articles to obtain information about COVID-19. Unsurprisingly, 35.3% of the postgraduate students used scientific websites and articles as a source of information about COVID-19 compared to 23.0% of the undergraduate students (Table 4).

**Table 4:** Source of information about COVID-19 among university students in Jordan by socio-demographic variables.

**Demographic variable**	**Category**	***N***	**Mass media**		**Internet and Social media**		**Scientific websites and articles**		**Others**	
			***N* (%)**	***P*-value**	***N* (%)**	***P*-value**	***N* (%)**	***P*-value**	***N* (%)**	***P*-value**
Overall		2,083	1,408 (67.6)		1,605 (77.1)		505 (24.2)		145 (7.0)	
Age	18–19.9 years	498	328 (65.9)	0.567	379 (76.1)	0.819	110 (22.1)	0.000	33 (6.6)	0.842
	20–24.9 years	1,304	892 (68.4)		1,007 (77.2)		297 (22.8)		94 (7.2)	
	≥25 years	281	188 (66.9)		219 (77.9)		98 (34.9)		18 (6.4)	
Gender	Female	1,572	1,080 (68.7)	0.058	1,187 (75.5)	0.003	348 (22.1)	0.000	87 (5.5)	0.000
	Male	511	328 (64.2)		418 (81.8)		157 (30.7)		58 (11.4)	
University	Northern universities	551	371 (67.3)	0.186	428 (77.7)	0.523	161 (29.2)	0.006	37 (6.7)	
	Middle universities	1,477	1,006 (68.1)		1,138 (77.0)		333 (22.5)		107 (7.2)	
	Southern universities	55	31 (56.4)		39 (70.9)		11 (20.5)		10 (18.7)	
College	Engineering	415	271 (65.3)	0.305	350 (84.3)	0.000	109 (26.3)	0.000	39 (9.4)	0.165
	Medical sciences	535	351 (65.6)		415 (77.6)		177 (33.1)		33 (6.2)	
	Agriculture and general sciences	376	262 (69.7)		292 (77.7)		90 (23.9)		22 (5.9)	
	Human sciences	757	524 (69.2)		548 (72.4)		129(17.0)		51 (6.7)	
Education level	Undergraduate	1,879	1,270 (67.6)	0.987	1448 (77.1)	0.974	433 (23.0)	0.000	136 (7.2)	0.132
	Postgraduate	204	138 (67.6)		157 (77.0)		72 (35.3)		9 (4.4)	
Accommodation type	Villa	106	69 (65.1)	0.711	80 (75.5)	0.020	32 (30.2)	0.260	11 (10.4)	0.383
	Flat	798	530 (66.4)		642 (80.5)		203 (25.4)		59 (7.4)	
	House	1,153	791 (68.6)		866 (75.1)		265 (23.0)		74 (6.4)	
	Others	26	18 (69.2)		17 (65.4)		5 (19.2)		1 (3.8)	
Place of residence	City	1,642	1,096 (66.7)	0.265	1,289 (78.5)	0.007	407 (24.8)	0.096	116 (7.1)	0.873
	Village	390	277 (71.0)		282 (72.3)		92 (23.6)		25 (6.4)	
	Others	51	35 (68.6)		34 (66.7)		6 (11.8)		4 (7.8)	

## Discussion

To the best of our knowledge, only one previously published study assessed the knowledge of Jordanian university students about COVID-19. However, the study only involved undergraduate students of Mutah University which is a government university located at the south of Jordan (17). The current study comprehensively assessed the knowledge and source of information about COVID-19 among postgraduate and undergraduate students from different fields of study in different government and private universities distributed over three zones: the north, middle and south of Jordan. The overall student COVID-19 knowledge score was 80.1%, indicating that most students were knowledgeable about this pandemic. This was expected because the survey was conducted just 1 d after the government-initiated lockdown of Jordan to control the COVID-19 pandemic, and 17 d after the first confirmed case of COVID-19 was reported in this country. Since then, the number of cases slowly increased during the conduct of the study. The present results are similar to those reported by Clements (18) who indicated that the average public knowledge score of US residents 2 months after illness began in the USA was 80%. However, the knowledge score reported in the current study is lower than that reported by Zhong et al. (19) who found that the overall knowledge score was 90% among Chinese residents during the rapid rise period of COVID-19 cases in Hubei Province, and Erfani et al. (20) who found that the average public knowledge of Iranians was 90% regarding general characteristics of COVID-19, and 85% regarding the mode of transmission and categories of people at high risk of COVID-19. On the other hand, Bhagavathula et al. (21) reported that a significant proportion of healthcare workers displayed poor knowledge about COVID-19 infection, particularly its transmission and incubation period.

The reasonably high knowledge score among Jordanian students likely resulted from their exposure to government information about COVID-19 which occurred before application of the quarantine. Furthermore, the overwhelming news reports about COVID-19, and the WHO characterization of the disease as a pandemic due its high pathogenicity and transmissibility (3) might also have increased the students' knowledge of COVID-19. It was observed that most students obtained their information about COVID-19 from the internet and social media as well as mass media including TV. Similarly, Alzoubi et al. (17) stated that social media was the most common source of information for Mutah university students. However, in the current study, medical sciences and postgraduate students, who were the most knowledgeable groups, used scientific websites and articles significantly more than their counterparts. Thus, the knowledge category was significantly associated with the major discipline and level of education. Unsurprisingly, other variables had no significant effect on the knowledge of students since the disease is considered as a serious threat worldwide.

Except for the type of SARS-Cov-2 viral genetic material and mortality rate of infection, 71–99.4% of students correctly answered the questions in the general knowledge of COVID-19. It is worth mentioning that the lower knowledge scores were related to the questions that required deep knowledge. About 72% of students were aware that there is no vaccine for COVID-19 and that antibiotics are not effective for its treatment. At the time of writing this report, there were no FDA approved vaccines or drugs for the prevention or treatment of COVID-19. Consequently, the current management of the illness includes prevention of disease by applying control measures and supportive care of infected patients by providing supplemental oxygen and mechanical ventilation (22). These preventive measures were adapted from previous outbreaks including the SARS 2003 epidemic (12).

Students also were knowledgeable regarding transmission mode and people at high risk of COVID-19 with a range of correct answers from 60.1 to 94.7%. Students showed extensive knowledge of the actual route of COVID-19 transmission such as saliva and nasal drip during talking or coughing and sneezing by infected individuals, kissing and shaking hands with SARS-COV-2 carriers, handling a patient's objects and materials as well as touching contaminated surfaces. This indicated that students were knowledgeable of these routes and could take steps to avoid getting sick. A major portion of the students knew that COVID-19 is not transmitted by sexual routes, consuming food or through the air. Ong et al. (23) pointed out that airborne transmission has not occurred in an analysis of approximately 75,500 cases in China.

About 40% of students believed that children under 5 years of age are at high risk of developing COVID-19. However, based on the reports of WHO (3) and CDC (1, 5), children are rarely infected and when this occurs it is generally with mild symptoms. The European Center of Disease Prevention and Control (24) reported that children under 10 years old represent a very small proportion (1%) of COVID-19 cases. On the other hand, the majority of students were aware that the elderly and persons with co-morbidities are at high risk of COVID-19. It was proven that people over 60 years old and those at all ages with underlying conditions; such as diabetes, hypertension, cardiovascular disease, cancer and chronic respiratory disease are at the highest risk of severe COVID-19 illness and even death (25).

The vast majority of students (>90%) were aware of the most common symptoms of COVID-19 such as fever, dry cough and shortness of breath. In contrast, large proportions of students (55.7–71.1%) were not knowledgeable about the less common symptoms such as weakness, rhinorrhea, vomiting and diarrhea. WHO (25) reported that the typical symptoms of COVID-19 include fever (87.9%), dry cough (67.7%), fatigue (38.1%), sputum production (33.4%), shortness of breath (18.6%), myalgia (14.8%), sore throat (13.9%), headache (13.6%), chills (11.4%), vomiting (5.0%), nasal congestion (4.8%), diarrhea (3.7%), and hemoptysis (0.9%) plus conjunctival congestion (0.8%) based on 56,000 laboratory-confirmed COVID-19 cases. Similarly, ECDC (24) reported that fever, dry cough, sore throat and general weakness were the most common symptoms of COVID-19 in 14,000 cases from 13 countries in Europe.

In the current study, 79.2–88.3% of the students were knowledgeable that pneumonia and bronchitis are complications of COVID-19. However, about 55.2% of students were aware that the disease may cause organ failure, and only 4.6% knew that sepsis is one of the COVID-19 complications. These low values could be because sepsis and multi-organ failure occur in severe COVID-19 cases which contribute to only 4% of infected people (3, 24). These complications can lead to a high mortality rate among infected persons and the vast majority of students (91.2%) in the current study recognized that severe respiratory failure could lead to death.

The WHO (12) expected that SARS-COV would not be the last emerging novel virus and it was followed by influenza A, H1N1, MERS, Ebola, Zika, and SARS-COV-2 viruses during the last two decades of the 21st century. Further, it is also expected that new viral diseases will evolve in the future at higher rate than at this period in time. Therefore, more fundamental information about viruses should be made available to the public facilitating the identification of risk factors for these diseases which should enable communities to deal with future emerging viral infections effectively and rapidly (26). Based on the results of the current study, it is suggested that public health authorities in collaboration with universities continuously implement health education programs about viral infections and other infectious diseases to university students through a required credit course during their studies, particularly those in non-medical programs to enhance their knowledge regarding these diseases so that they might directly engage in the implementation of protective health measures to contain infectious diseases such as the COVID-19 pandemic.

## Conclusions

This study showed good knowledge of COVID-19 among 2083 postgraduate or undergraduate students from different universities in Jordan with an overall knowledge score of 80.1%. The students showed extensive knowledge of most questions about general information, transmission route, symptoms, complications and people at high risk of COVID-19. However, the students' knowledge was significantly affected by the college of study and education level where medical and postgraduate students had the highest levels of knowledge. The least common symptoms (such as vomiting and diarrhea) and complications (such as weakness and sepsis) were not well recognized by students. Therefore, these results could help in assessing the actual situation to apply educational health programs and measures.

## Data Availability Statement

The raw data supporting the conclusions of this article will be made available by the authors, without undue reservation.

## Ethics Statement

The studies involving human participants were reviewed and approved by the Institutional Review Board (IRB) committee of the Hashemite University. The patients/participants provided their written informed consent to participate in this study.

## Author Contributions

AO and IA designed the study, interpreted the results, and collected test data. AO, IA, HS, and RH wrote the manuscript.

## Conflict of Interest

The authors declare that the research was conducted in the absence of any commercial or financial relationships that could be construed as a potential conflict of interest.

## References

[1] CDC Coronavirus (COVID-19). (2020). Available online at: 10.1016/S0140-6736(20)30673-5 (accessed April 8, 2020). 10.1016/S0140-6736(20)30673-5

[2] Gao J Tian Z Yang X. Breakthrough: chloroquine phosphate has shown apparent efficacy in treatment of COVID-19 associated pneumonia in clinical studies. Biosci Trends. (2020) 14:72–3. 10.5582/bst.2020.01047 32074550

[3] WHO Coronavirus Disease (COVID-19) Pandemic. (2020). Available online at: https://www.who.int/emergencies/diseases/novel-coronavirus-2019 (accessed May 7, 2020).

[4] Jordanian Ministry of Health COVID-19. (2020). Available online at: https://corona.moh.gov.jo/ar (Accessed April 10, 2020).

[5] CDC. Severe outcomes among patients with coronavirus disease 2019 (COVID-19) — United States, February 12–March 16. Morb. Mortal. Wkly. Rep. 69:343–6. 10.15585/mmwr.mm6912e2 32214079 PMC7725513

[6] Wang M Cao R Zhang L Yang X Liu J Xu M. Remdesivir and chloroquine effectively inhibit the recently emerged novel coronavirus (2019-nCoV) *in vitro*. Cell Res. (2020) 30:269–71. 10.1038/s41422-020-0282-0 32020029 PMC7054408

[7] Lu H. Drug treatment options for the 2019-new coronavirus (2019-nCoV) (2020). Biosci Trends. (2020) 14:69–71. 10.5582/bst.2020.01020 31996494

[8] Gautret P Lagier J-C Parola P Hoang V Meddeb L Mailhe M. Hydroxychloroquine and azithromycin as a treatment of COVID-19: results of an open-label non-randomized clinical trial. Int J Antimicrob Agents. 10.1016/j.ijantimicag.2020.105949. [Epub ahead of print]. 32205204 PMC7102549

[9] Armocida B Formenti B Ussai S Palestra F Missoni E. The Italian health system and the COVID-19 challenge. Lancet Public Health. 5:E253. (2020) 10.1016/S2468-2667(20)30074-8 32220653 PMC7104094

[10] Gonzalez RI Munoz F Moya PS Kiwi M Is a COVID19 quarantine justified in Chile or USA right now? (2020). arXiv:2003.10879 [Preprint]. 10.1101/2020.03.23.20042002

[11] Rewar S Mirdha D Rewar P. Treatment and prevention of pandemic H1N1 influenza. Ann Glob Health. (2015) 81:645–53. 10.1016/j.aogh.2015.08.014 27036721

[12] WHO Chapter 5: SARS: Lessons From a New Disease. (2003). Available online at: https://www.who.int/whr/2003/chapter5/en/index5.html (accessed May 7, 2020).

[13] Pappaioanou M Gramer M. Lessons from pandemic H1N1 2009 to improve prevention, detection, and response to influenza pandemics from a One Health perspective. ILAR J. (2010) 51:268–80. 10.1093/ilar.51.3.268 21131728 PMC7314042

[14] Bedford J Enria D Giesecke J Heymann DL Ihekweazu C Kobinger G. COVID-19: towards controlling of a pandemic. Lancet. (2020) 395:1015–8. 32197103 10.1016/S0140-6736(20)30673-5 PMC7270596

[15] Maffetone PB Laursen PB. The perfect storm: COVID-19 pandemic meets overfat pandemic. Front. Public Health. (2020) 8:135. 10.3389/fpubh.2020.00135 32391307 PMC7190793

[16] Raosoft Inc RaoSoft® Sample Size Calculator. (2004). Available online at: http://www.raosoft.com/samplesize.html (accessed March 18, 2020).

[17] Alzoubi H Alnawaiseh N Al-Mnayyis A Abu-Lubada M Aqel A Al-Shagahin H COVID-19 - knowledge, attitude and practice among medical and non-medical university students in Jordan J. Pure Appl Microbiol. (2020) 14:17–24. 10.22207/JPAM.14.1.04

[18] Clements JM. Knowledge Behaviors Toward COVID-19 Among U.S. Residents During the Early Days of the Pandemic. Available online at: https://www.medrxiv.org/content/10.1101/2020.03.31.20048967v1 (accessed April 8, 2020). 10.1101/2020.03.31.20048967 PMC7212816 32369759

[19] Zhong B Luo W Zhang QQ Liu XG Li WT Li Y Knowledge, attitudes, and practicestoward COVID-19 among Chinese residents during rapid rise period of COVID-19 outbreak: aquick online cross-sectional survey. Int J Biol Sci. (2020) 16:1745–52. 10.7150/ijbs.45221 32226294 PMC7098034

[20] Erfani A Shahriarirad R Ranjbar K Mirahmadizadeh A Moghadami M Knowledge, attitude and practice toward the novel coronavirus (COVID-19) outbreak: A population-based survey in Iran. Bull World Health Organ (2020). Available online at: https://www.who.int/bulletin/online_first/COVID-19/en/ (accessed April 8, 2020). 10.2471/BLT.20.256651

[21] Bhagavathula AS Aldhaleei WA Rahmani J Mahabadi MA Bandari DK Novel Coronavirus (COVID-19) Knowledge and Perceptions: A Survey of Healthcare Workers. (2020). Available online at: https://www.medrxiv.org/content/10.1101/2020.03.09.20033381v2 (accessed April 8, 2020). 10.1101/2020.03.09.20033381

[22] U.S. Food and Drug Administration Coronavirus Disease 2019 (COVID-19). (2020). Available online at: https://www.fda.gov/emergency-preparedness-and-response/counterterrorism-and-emerging-threats/coronavirus-disease-2019-covid-19 (accessed April 8, 2020).

[23] Ong SWX Tan YK Chia PY Lee TH Ng OT Wong MSY Air, surface environmental, and personal protective equipment contamination by severe acute respiratory syndrome coronavirus 2 (SARS-CoV-2) from a symptomatic patient. JAMA. (2020) 4:E1–E3. 10.1001/jama.2020.3227 PMC7057172 32129805

[24] ECDC COVID-19. (2020). Available online at: https://www.ecdc.europa.eu/en/covid-19-pandemic (accessed April 8, 2020).

[25] WHO. Report of the WHO-China Joint Mission on Coronavirus Disease 2019 (COVID-19). (2020). Available online at: https://www.who.int/publications-detail/report-of-the-who-china-joint-mission-on-coronavirus-disease-2019-(covid-19) (accessed May 7, 2020).

[26] Grubaugh ND Ladner JT Lemey P Pybus OG Rambaut A Holmes EC. Tracking virus outbreaks in the twenty-first century. Nat Microbiol. (2019) 4:10–9. 10.1038/s41564-018-0296-2 30546099 PMC6345516

